# Application of an acoustofluidic perfusion bioreactor for cartilage tissue engineering

**DOI:** 10.1039/c4lc00956h

**Published:** 2014-10-01

**Authors:** Siwei Li, Peter Glynne-Jones, Orestis G. Andriotis, Kuan Y. Ching, Umesh S. Jonnalagadda, Richard O. C. Oreffo, Martyn Hill, Rahul S. Tare

**Affiliations:** a Centre for Human Development , Stem Cells and Regeneration , Faculty of Medicine , University of Southampton , Southampton SO16 6YD , UK . Email: rt2@soton.ac.uk ; Fax: +44 2381 204221 ; Tel: +44 (0)2381 205257; b Engineering Sciences , Faculty of Engineering and the Environment , University of Southampton , Southampton SO17 1BJ , UK; c Institute of Lightweight Design and Structural Biomechanics , Vienna University of Technology , Gusshausstrasse 27-29 A-1040 , Vienna , Austria; d nCATS , Faculty of Engineering and the Environment , University of Southampton , Southampton SO17 1BJ , UK

## Abstract

Cartilage grafts generated using conventional static tissue engineering strategies are characterised by low cell viability, suboptimal hyaline cartilage formation and, critically, inferior mechanical competency, which limit their application for resurfacing articular cartilage defects. To address the limitations of conventional static cartilage bioengineering strategies and generate robust, scaffold-free neocartilage grafts of human articular chondrocytes, the present study utilised custom-built microfluidic perfusion bioreactors with integrated ultrasound standing wave traps. The system employed sweeping acoustic drive frequencies over the range of 890 to 910 kHz and continuous perfusion of the chondrogenic culture medium at a low-shear flow rate to promote the generation of three-dimensional agglomerates of human articular chondrocytes, and enhance cartilage formation by cells of the agglomerates *via* improved mechanical stimulation and mass transfer rates. Histological examination and assessment of micromechanical properties using indentation-type atomic force microscopy confirmed that the neocartilage grafts were analogous to native hyaline cartilage. Furthermore, in the *ex vivo* organ culture partial thickness cartilage defect model, implantation of the neocartilage grafts into defects for 16 weeks resulted in the formation of hyaline cartilage-like repair tissue that adhered to the host cartilage and contributed to significant improvements to the tissue architecture within the defects, compared to the empty defects. The study has demonstrated the first successful application of the acoustofluidic perfusion bioreactors to bioengineer scaffold-free neocartilage grafts of human articular chondrocytes that have the potential for subsequent use in second generation autologous chondrocyte implantation procedures for the repair of partial thickness cartilage defects.

## Abstract 2

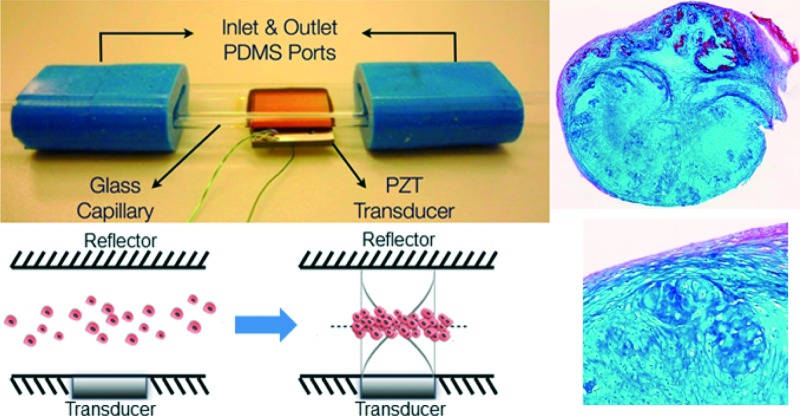
Bioengineering neocartilage grafts of human articular chondrocytes in a custom-built microfluidic perfusion bioreactor with integrated ultrasound standing wave trap.

## 1. Introduction

Osteoarthritis (OA), the most common form of arthritis in the western world, is a degenerative joint disease that is characterised by the deterioration and progressive loss of articular cartilage. Early stages of OA are associated with the development of clefts and fissures in the articular cartilage surface that resemble partial thickness defects or chondral defects. Partial thickness defects are unable to heal spontaneously because the lesions do not extend into the marrow spaces of the subchondral bone and cannot gain access to the bone marrow skeletal stem cells to promote repair.^1^ It is therefore critical to repair these defects in the early stages of OA because, if left untreated, the fibrillated lesions typically grow larger and deeper over time, and contribute to progressive articular cartilage degeneration and joint immobilisation. Currently, there are no effective pharmacological agents that are able to promote comprehensive healing of articular cartilage defects.

Surgical interventions for functional restoration of articular cartilage defects include reparative bone marrow stimulation techniques such as abrasion arthroplasty, drilling, microfracture, and restorative approaches such as autologous chondrocyte implantation (ACI), osteochondral auto/allografts, periosteal/perichondral grafts.^2^ Although these interventions provide symptomatic relief and improve joint function temporarily, to date, no technique has been completely successful in restoring/regenerating damaged articular cartilage to its native state. This is because the repair tissue that is generated is often fibrocartilaginous in nature, and therefore lacks the mechanical competency of hyaline articular cartilage. Moreover, inability of the implanted graft/fibrocartilaginous repair tissue to integrate with the surrounding native cartilage contributes to graft failure and further degeneration of the joint.

Attempts to improve the outcomes of cell-based transplantation methods, such as ACI, have therefore focused on the application of autologous chondrocytes seeded onto collagen scaffolds, in matrix-induced ACI, and three-dimensional (3-D) cartilaginous constructs, in second (II) generation ACI, for the repair of articular cartilage defects.^3–5^ This, in turn, has led to considerable interest in the development of effective scaffold-free and scaffold-based tissue engineering strategies for generating cartilage grafts. Scaffold-free modalities have been employed to stimulate chondrogenic differentiation and cartilage formation *via* cell–cell and cell–matrix interactions in high-density chondrospheres and multilayer articular chondrocyte sheets.^6,7^ In contrast to the scaffold-free modalities, which require cells to develop their own structure and matrix during development, strategies harnessing scaffolds provide cells with an effective 3-D framework that supports tissue assembly and growth. However, the successful application of biomaterials in tissue engineering, including cartilage bioengineering, requires careful consideration of their 3-D architecture, biofunctionality, biocompatibility, biomechanics, degradation rates and immunogenicity of the degradation products.^8,9^

Tissue grafts generated using conventional static tissue engineering strategies are often characterised by appreciable necrosis and suboptimal tissue formation due to poor nutrient mass transfer rates and oxygen diffusion in almost any graft site with diffusion distance more than 1 mm.^10^ To address some of the issues related to suboptimal tissue formation and cell viability in constructs generated using 3-D static culture techniques, tissue engineering strategies have increasingly applied bioreactors, which provide a closely monitored environment and, critically, biomechanical stimuli such as hydrodynamic shear stress, hydrostatic pressure and dynamic compression for optimal tissue growth.^11^ Bioreactors have been used to culture a wide range of cells, namely stem cells, chondrocytes, osteoblasts, keratinocytes, hepatocytes, cardiomyocytes and myofibroblasts, for a diverse array of applications including cartilage, bone, skin, liver and cardiovascular tissue engineering.^11,12^ In particular, perfusion flow bioreactors have been frequently used for cartilage tissue engineering due to their ability to enhance cartilage formation by chondrocytes and mesenchymal cell populations through the application of mechanical stimuli in the form of fluid flow-induced hydrodynamic shear stresses, and improvement of mass transfer rates of metabolites and oxygen.^13,14^

The safe use of ultrasound and its diverse diagnostic and therapeutic applications are widely acknowledged.^15–17^ Low intensity pulsed ultrasound has been demonstrated to accelerate the repair of damaged cartilage in a number of animal studies. Low intensity ultrasound typically refers to field intensities below 1 W cm^–2^ that are applied either in continuous or burst (pulsed) mode.^18,19^ A variation of low intensity ultrasound, referred to as low intensity diffuse ultrasound, involves scattering of acoustic waves generated by the transducer throughout the chamber.^20^ In the rat model of papain-induced knee osteoarthritis, the application of ultrasound was shown to enhance cartilage repair in the early stage of the disease, and arrest further deteriorative cartilage damage in the later stage of osteoarthritis.^21^ Daily low intensity pulsed ultrasound was demonstrated to have a significant positive effect on the repair of full thickness osteochondral defects created in the patellar grooves of rabbits.^22^ In a canine model, low intensity pulsed ultrasound enhanced the incorporation of autologous osteochondral plugs by improving the characteristics of the interface repair tissue and its integration with the adjacent cartilage.^23^

In the field of tissue engineering, ultrasound has predominantly been applied in the form of low intensity ultrasound to stimulate cells, and ultrasonic standing wave fields to generate acoustic traps, which can spatially manipulate cells, proteins and microbeads.^24^ Specifically for cartilage tissue engineering, the application of low intensity ultrasound to enhance chondrogenic differentiation of bone marrow mesenchymal stem cells and chondrocytes, cultured in a variety of 3-D environments, has been documented in a number of studies.^25–29^ However, ultrasonic cell trapping, a non-destructive and non-invasive cell manipulation technique,^30^ is a relatively less exploited application of ultrasound for cartilage tissue engineering. When a fluid containing a suspension of cells is exposed to an ultrasonic standing wave field in a chamber/trap, the acoustic radiation force arising from the scattering of the acoustic waves on the cells directs the motion (*i.e.* acoustophoresis) of cells typically to areas of minimum pressure, referred to as pressure nodes, and facilitates their aggregation into multicellular clusters.^31–33^ Furthermore, it is possible to drive ensembles of cells into geometric formations, including linear clusters and planar (2-D) sheets, by appropriately shaping the resonant wave field within the ultrasonic trap, and also levitate the multicellular aggregates away from the influence of the solid substrate.^34,35^

The present study applied a novel approach that combined bioreactor technology with ultrasonic cell trapping to bioengineer 3-D, scaffold-free neocartilage grafts of human articular chondrocytes in custom-built microfluidic perfusion bioreactors with integrated ultrasound standing wave traps (USWT). The neocartilage grafts were then examined for their potential to repair partial thickness chondral defects. The study has demonstrated the first successful application of the acoustofluidic perfusion bioreactor for bioengineering scaffold-free, hyaline cartilage-like explants of human articular chondrocytes. Following implantation into partial thickness chondral defects, the bioengineered explants generated hyaline cartilage-like repair tissue that integrated closely with the surrounding host articular cartilage and contributed to significant improvements to the tissue architecture within the defects. The neocartilage grafts, therefore, have the potential for application in restorative surgical procedures, such as II generation ACI, to repair early-stage articular cartilage damage and limit further cartilage degeneration.

## 2. Materials and methods

Most chemicals and reagents were purchased from Invitrogen/Life Technologies Ltd. (Paisley, UK) and Sigma-Aldrich (Gillingham, UK) unless specified otherwise.

### 2.1 Isolation of human articular chondrocytes (HACs)

Femoral head samples were obtained from 4 haematologically normal osteoarthritic individuals (3 female and 1 male, mean age: 80 years) following routine total hip replacement surgery for late-stage OA at Southampton General Hospital. Only tissue that would have been discarded was used in this study with approval of the Southampton and South West Hampshire Research Ethics Committee (ref no. 194/99/1 & 210/01). HACs were isolated by sequential enzymatic digestion of deep-zone articular cartilage pieces dissected from femoral heads, as explained previously.^36^ In brief, cartilage pieces were sequentially digested with 500 μg ml^–1^ trypsin-EDTA for 30 min, 1 mg ml^–1^ hyaluronidase for 15 min and 10 mg ml^–1^ collagenase B (Roche Diagnostics, Burgess Hill, UK) overnight on a rotating mixer at 37 °C. Isolated chondrocytes were cultured to confluence in monolayer cultures in α-MEM supplemented with 10% (v/v) FCS, 100 units ml^–1^ penicillin, 100 μg ml^–1^ streptomycin and 100 μM ascorbate 2-phosphate. Cultures were maintained in humidified atmosphere at 37 °C, 5% CO_2_ and 21% O_2_. Passage 1 cells were utilised for the experiments.

### 2.2 Fabrication of perfusion bioreactors with integrated USWT/acoustofluidic perfusion bioreactors and their application for generation of neocartilage grafts of HACs

**Fig. 1:**
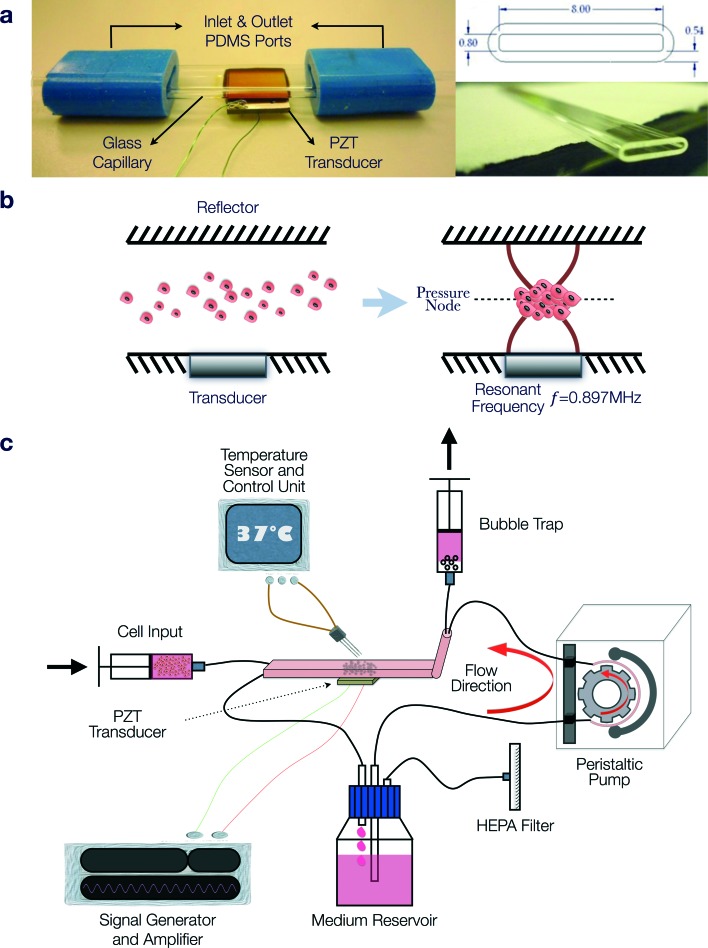
Application of the acoustofluidic perfusion bioreactor for the generation of neocartilage grafts. (a) The bioreactor was fabricated using a rectangular glass capillary (length 5 cm, ID 0.8 × 8 mm^2^, wall thickness 0.54 mm), polydimethylsiloxane (PDMS) connectors and a ceramic piezoelectric transducer (PZT), which was glued to the capillary. (b) Schematic diagram illustrating formation of the multicellular agglomerate in the resonant chamber. The transducer generated an ultrasonic standing wave field in the lumen of the capillary, with the upper glass surface acting as a reflector. At the resonant frequency (0.897 MHz) of the cavity, a half-wavelength mode was established with a pressure node in the centre of the chamber. When cells suspended in culture medium were introduced into the chamber, the acoustic radiation forces directed the cells to the pressure node, where they aggregated rapidly and eventually formed the multicellular agglomerate, which was levitated in the lumen of the chamber above the transducer. (c) Serum-free chondrogenic medium held in the reservoir was circulated by a peristaltic pump around a closed loop that included the resonant chamber, bubble trap and syringe pump used to introduce cells into the chamber. Wires soldered to the electrodes on the transducer were connected to a custom-made amplifier that was driven by a signal generator. Although activation of the ultrasound caused heating, at the ambient temperature (36 °C) of the system, the active region of the chamber reached a steady 37.0 ± 0.5 °C.

The acoustofluidic perfusion bioreactors were custom-built using commercially available rectangular glass capillaries (VitroCom 4608-100, CM Scientific, Silsden, UK) and polydimethylsiloxane (PDMS) connectors cast from acrylic moulds (Fig. 1a). The glass capillaries functioned efficiently as highly resonant cavities,^37–39^ and it was possible to autoclave them along with the non toxic PDMS. A set of 3 resonant chambers were fabricated from the rectangular glass capillaries (length 5 cm, ID 0.8 × 8 mm^2^, wall thickness 0.54 mm) and a ceramic piezoelectric transducer (PZT) (Ferroperm PZ26, Kvistgaard, Denmark; 10 × 8 × 1 mm^3^) was glued (Epoxy 353ND, Epotek, Billerica, MA, USA) to each chamber. Wires soldered to the electrodes on the transducer were connected to a custom-made amplifier (LT1210 IC, RS Components, Corby, UK) that was driven by a signal generator (TG200, TTi, Huntingdon, UK).

The transducer generated an ultrasonic standing wave field in the lumen of the capillary, with the upper glass surface acting as a reflector. An impedance spectrum was used in conjunction with a transfer impedance model to predict the resonant frequencies of the system.^40,41^ The half-wavelength resonance of the cavity was found at 897, 899 and 902 kHz for the three devices respectively. The half-wavelength resonance had a pressure node in the centre of the chamber and was observed to i) promote the formation of a 3-D multicellular agglomerate by rapid aggregation at the pressure node of HACs introduced into the chamber, and ii) levitate the agglomerate in the lumen of the chamber above the transducer away from the influence of the solid substrate (Fig. 1b). The voltage drop method was used to assess the acoustic pressure amplitude;^31^ a range of locations over the transducer were examined with average acoustic pressure amplitude of 17 ± 5.1 kPa V^–1^. The generator was adjusted to create a voltage of 10 V_pp_ (peak-to-peak voltage) across the transducer (average over sweep range). In addition to the primary potential energy gradients found perpendicular to the transducer, smaller forces caused by gradients in the kinetic energy density parallel to the transducer created localised trapping forces.^42^ These forces assisted agglomerate formation (aided by the secondary inter-particle forces) and also held the agglomerate against the perfusion flow. It was found that a typical agglomerate of HACs could withstand linear fluid velocities of up to approximately 1 mm s^–1^, thus trapping the levitated agglomerate against the flow of continuous perfusion.

Serum free chondrogenic culture medium held within a reservoir was circulated around a closed loop by a peristaltic pump (403U/VM2, Watson-Marlow, Falmouth, UK) and Marprene tubing (505DZ/RL, Fisher Scientific, UK; ID 0.8 mm) at a rate of 1.32 ml h^–1^ (Fig. 1c). The serum-free chondrogenic medium was composed of α-MEM supplemented with 10 ng ml^–1^ rhTGF-β3 (PeproTech, London, UK), 100 μM ascorbate-2-phosphate, 10 nM dexamethasone and 1X ITS liquid supplement (10 μg ml^–1^ insulin, 5.5 μg ml^–1^ transferrin and 5 ng ml^–1^ selenite premix). The loop included the resonant chamber connected to the tubing *via* PDMS connectors and a bubble trap in close proximity to the chamber to prevent bubbles disrupting the levitated agglomerate. A syringe pump enabled excess bubbles to be extracted from the bubble trap; during the experiment this was run at a continuous rate of 0.02 ml h^–1^. The optimum CO_2_ concentration was maintained by preconditioning the chondrogenic medium in a standard CO_2_ incubator for 24 h to allow gaseous equilibrium and creating a 5% CO_2_ atmosphere in the space above the culture medium in the reservoir through introduction of the gas *via* a HEPA filter with 0.22 μm pore size.

Another syringe pump was used to introduce cells into the chamber *via* a dedicated inlet. A suspension of HACs containing 1 × 10^6^ cells was introduced at a rate of 1 ml min^–1^ with the ultrasound active. The perfusion peristaltic pump was activated once stable agglomerates were formed. The system was placed within a poly(methyl methacrylate)/PMMA box covered with expanded polystyrene sheets for thermal insulation and a custom heating controller circulated hot air to maintain the ambient temperature at 36 °C. Although activation of the ultrasound was found to cause heating, at the ambient temperature, the active region of the chamber reached a steady 37.0 ± 0.5 °C. The multicellular agglomerates were cultured within the acoustofluidic perfusion bioreactors over a period of 21 days in serum-free chondrogenic medium to promote cartilage formation. At the end of the culture period, two grafts were labelled with Cell Tracker^™^ Green and Ethidium homodimer-1, fixed in 4% paraformaldehyde (PFA) overnight at 4 °C and used for histological analysis; three grafts were used for determination of biomechanical properties and two grafts were harvested for implantation into partial thickness chondral defects.

### 2.3 Assessment of cell viability

Cell viability in the day 21 neocartilage grafts was examined by ‘live-dead’ staining using a combination of CellTracker^™^ green/CTG CMFDA (5-chloromethylfluorescein diacetate) and Ethidium homodimer-1/EH-1. Harvested samples were washed in PBS and incubated in 1 ml of CTG (10 μg ml^–1^) and EH-1 (5 μg ml^–1^) solution for 45 min in a humidified atmosphere at 37 °C, 5% CO_2_ and 21% O_2_. Samples were then fixed in 4% PFA overnight at 4 °C. Viable cells (green) and necrotic cells (red) were visualised in paraffin wax sections of the constructs by fluorescence microscopy using a Zeiss Axiovert 200 inverted fluorescence microscope (Carl Zeiss, Cambridge, UK) and imaged using a CCD camera with Axiovision software.

### 2.4 *Ex vivo* organ culture partial thickness cartilage defect model

Near full-thickness articular cartilage pieces (1 × 1 cm^2^, 2 mm thick) were dissected from healthy non load-bearing regions of human femoral heads. A partial thickness defect (~2 × 2 mm^2^, 1 mm deep) was created in each articular cartilage piece with a sterile drill bit, taking extreme care to avoid full penetration of the cartilage. One neocartilage graft was implanted into each defect and the neocartilage graft-host cartilage construct was then placed on a Millipore filter insert (hydrophilic polytetrafluoroethylene/PTFE membrane, 0.4 μm pore size, 6-well plate configuration) and cultured at air–liquid interface in a humidified atmosphere at 37 °C, 5% CO_2_ and 21% O_2_ for 16 weeks. Pieces of articular cartilage with empty defects cultured similarly for 16 weeks served as controls. The samples were harvested at 16 weeks, fixed in 4% PFA overnight at 4 °C and processed for histological analysis.

### 2.5 Processing, embedding and section cutting

PFA-fixed samples were processed through graded ethanol (50–100%) and histoclear (100%) prior to embedding in paraffin wax. Sequential sections (7 μm) were cut on the microtome and mounted on glass slides for histological and immunohistochemical staining. Images were captured with Olympus dotSlide virtual microscopy system (Olympus, Southend-on-Sea, UK).

### 2.6 Alcian blue and Sirius red staining

Sections were stained with Alcian blue 8GX (5 mg ml^–1^ in 1% (v/v) glacial acetic acid) and Sirius red F3B (10 mg ml^–1^ in saturated picric acid) following nuclear staining with Weigert's haematoxylin.

### 2.7 Immunohistochemistry

After quenching endogenous peroxidase activity with 3% (v/v) H_2_O_2_ and blocking with 10 mg ml^–1^ BSA in PBS, sections were incubated with relevant primary antisera (diluted appropriately using 10 mg ml^–1^ BSA in PBS) at 4 °C overnight. This was followed by hour-long incubation each with the appropriate biotinylated secondary antibody and ExtrAvidin®-Peroxidase. Visualisation of the immune complex was undertaken using the avidin–biotin method linked to peroxidase and AEC (3-amino-9-ethylcarbazole), resulting in a reddish brown reaction product. Negative controls (omission of the primary antisera) were included in all immunohistochemistry procedures. No staining was observed in the sections used as negative controls. Sections were counter-stained with Alcian blue. The anti-SOX9 (AB5535, Millipore, Watford, UK) antibody was used at dilution of 1 : 150 following the antigen retrieval procedure, which involved heating sections in 0.01 M citrate buffer (pH 6.0) for 5 minutes before the application of the standard immunohistochemistry procedure. Sections were treated with type I hyaluronidase at 37 °C for 20 min in order to unmask the collagen fibres and render them accessible for immunostaining with the antibodies against the three types of collagen. The anti-collagen type I antibody (LF68 from Dr Larry Fisher, NIH, USA), anti-collagen type II and anti-collagen type X antibodies (cat. no. 234187 and 234196, both sourced from Millipore, Watford, UK) were used at dilutions of 1 : 1000, 1 : 500 and 1 : 100, respectively.

### 2.8 Indentation-type Atomic Force Microscopy (IT-AFM)

As described previously,^43^ cantilever-based IT-AFM with a micrometre-sized spherical tip was used to measure the microscale elastic moduli of day 21 neocartilage grafts and full-thickness articular cartilage pieces, dissected from femoral heads of patients (2 female and 1 male, average age: 77 years) who had suffered a fracture in the neck of femur. In brief, hard borosilicate glass spheres (diameter: 10 μm; 02715-AB, SPI Supplies, West Chester, PA, USA) were glued onto tipless rectangular silicon AFM cantilevers (spring constant: 6.11 N m^–1^; type All In One-TL, BudgetSensors, Sofia, Bulgaria) for probing the samples. Force volume maps of load–displacement curves (16 × 16 *i.e.* 256 load–displacement curves) were recorded in a regular grid over the sample surface. Each individual set of data consisted of load–displacement curves recorded at a rate of half full loading cycles per second in a sample area of 10 μm × 10 μm. A maximum cantilever deflection of 200 nm coupled with a maximum applied load of 1.2 μN was used for the measurement of all samples. The samples were fixed on a glass slide and submerged in PBS during the measurements, which were performed at room temperature using a MFP-3D AFM (Oxford Systems, Asylum Research, Santa Barbara, CA, USA). Values for microscale elastic moduli of the samples were calculated from the unloading curves obtained from IT-AFM, ensuring that the displacement data did not contain irreversible (*i.e.* plastic) deformation. Statistical analysis was performed using Mann–Whitney *U* test. Results were considered significant if the probability of occurrence by random chance alone was less than 5% (*i.e. p* < 0.05).

## 3. Results

### 3.1 Examination of day 21 grafts for assessment of cell viability, cartilage formation and micromechanical properties

**Fig. 2:**
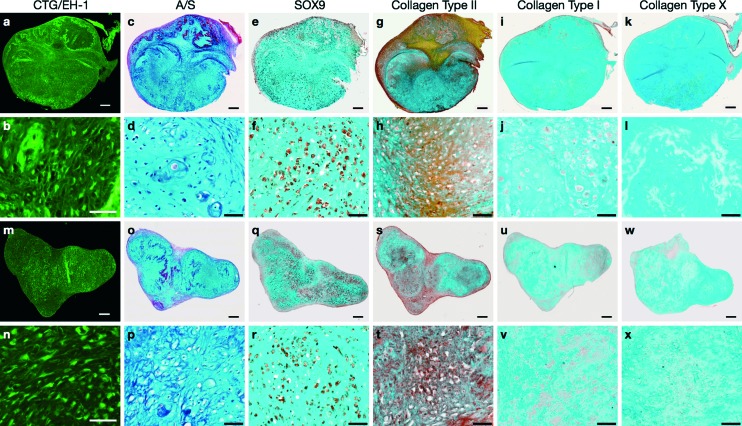
Cell viability and chondrogenic differentiation in neocartilage grafts of HACs harvested from the acoustofluidic perfusion bioreactors following the 21 day culture period in chondro inductive medium. (a, b, m, n) Sections of the grafts labelled with Cell Tracker^™^ Green and Ethidium homodimer-1 (live-dead staining) exhibited metabolically viable cells that were stained fluorescent green and absence of necrotic cells. (c, d, o, p) Histological sections of the grafts stained with Alcian blue and Sirius red displayed hyaline cartilage-like tissue composed of chondrocytes in lacunae embedded in proteoglycan and collagen-rich extracellular matrix. Chondrogenic differentiation was examined by immunostaining sequential sections of the grafts with antibodies against the phenotypic proteins and confirmed by the presence of (e, f, q, r) SOX-9 in chondrocytes and (g, h, s, t) type II collagen in the extracellular matrix. (i, j, u, v) Negligible staining was observed for type I collagen, while (k, l, w, x) lack of staining for type X collagen indicated absence of chondrocyte hypertrophy in the neocartilage grafts. Scale bars for low and high magnification images represent 200 μm and 50 μm respectively.

3-D membrane-like constructs (measuring ~2.5 × 2.5 mm^2^, 0.2 mm thick) were harvested from the acoustofluidic perfusion bioreactors following the 21 day culture period. The constructs were labelled with Cell Tracker^™^ Green and Ethidium homodimer-1 to determine metabolically viable and necrotic cells, respectively. Intense green staining coupled with absence of red staining in the sections indicated robust cell viability and negligible cell necrosis within the constructs (Fig. 2a, b, m, n). Sequential sections of the constructs were stained using a range of routine histological and immunohistochemical techniques for analysis of cartilage formation and chondrogenic differentiation. Histological examination of the sections revealed hyaline cartilage-like tissue composed of numerous chondrocytes within lacunae embedded in dense extracellular matrix constituted by proteoglycans and collagen, which were stained with Alcian blue and Sirius red, respectively (Fig. 2c, d, o, p). Extensive expression of SOX-9, the key marker of chondrogenesis, was observed in most cells of the constructs (Fig. 2e, f, q, r), along with immunostaining for type II collagen, the hyaline cartilage-specific collagen, in areas of collagenous matrix (Fig. 2g, h, s, t). Further immunohistochemical analysis of the constructs confirmed negligible expression of type I collagen, a key constituent of fibrocartilage and bone matrix (Fig. 2i, j, u, v), and type X collagen, a phenotypic marker of hypertrophic cartilage (Fig. 2k, l, w, x), in the extracellular matrix.

**Fig. 3:**
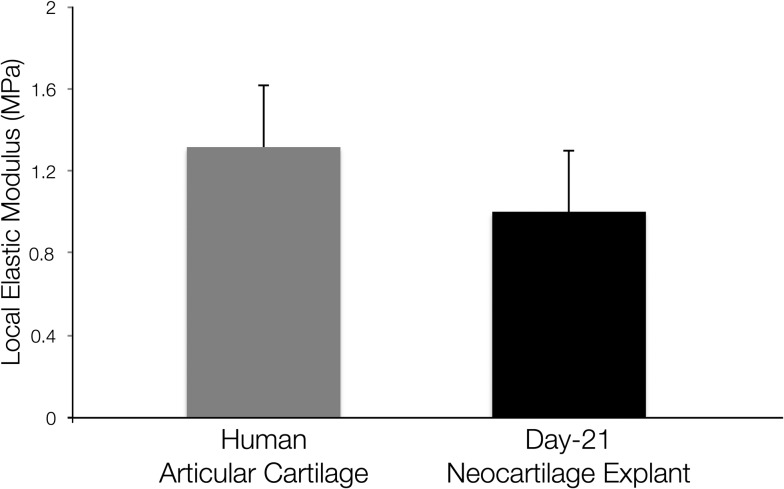
Micromechanical properties of human articular cartilage and neocartilage grafts generated using the acoustofluidic perfusion bioreactors were determined by IT-AFM. The difference between values for microscale elastic moduli of full thickness human articular cartilage samples and day 21 neocartilage grafts was not statistically significant. Values are expressed as mean ± SD, *n* = 3 in each group.

The micromechanical properties of the neocartilage constructs were compared with full thickness human articular cartilage. The values for microscale elastic moduli of the neocartilage constructs and full thickness human articular cartilage samples determined by IT-AFM were found to be comparable (Fig. 3).

### 3.2 Assessment of the quality of the repair tissue and its integration with the native articular cartilage matrix in the partial thickness chondral defect

**Fig. 4:**
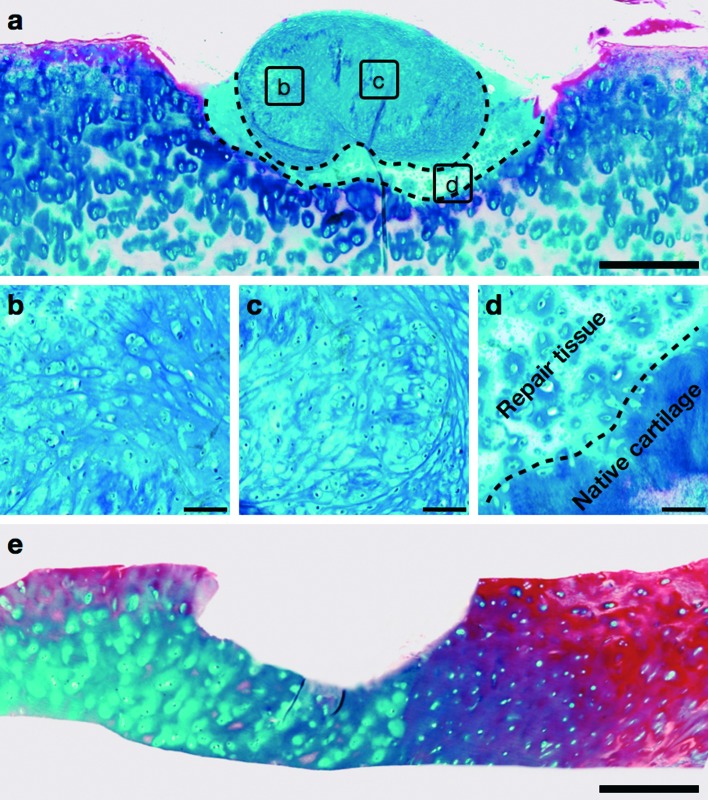
Generation of repair tissue after 16 weeks of implantation of the neocartilage graft into the partial thickness defect created in the host human articular cartilage. (a–c) The defect region in the host articular cartilage exhibited the presence of hyaline cartilage-like graft and repair tissue (demarcated with dashed lines in a), composed of numerous chondrocytes embedded in dense proteoglycan-rich matrix stained with Alcian blue. (d) The hyaline cartilage-like repair tissue fused closely with the host articular cartilage; the dashed line demarcated the boundary between the repair tissue and native cartilage. (e) Representative image of an empty defect demonstrating absence of cartilage regeneration after 16 weeks of culture under identical conditions. Scale bars represent 500 μm in a and e, and 50 μm in b–d.

The histology of the repair tissue, generated after 16 weeks of implantation of the neocartilage construct into the partial thickness defect of the host articular cartilage, was examined in sections stained with Alcian blue and Sirius red. Following 16 weeks of co-culture, the implanted graft integrated into the host articular cartilage and maintained the hyaline cartilage-like morphology, characterised by numerous chondrocytes embedded in dense proteoglycan matrix stained with Alcian blue (Fig. 4a–c). The repair tissue (demarcated by dashed lines in Fig. 4a), generated by the graft upon implantation into the defect, was analogous to hyaline cartilage and demonstrated the presence of chondrocytes and proteoglycan-rich extracellular matrix. Moreover, the repair tissue exhibited close continuous attachment with the host cartilage and no gaps were observed between the newly synthesized cartilage and host articular cartilage along the boundary of the defect (demarcated by dashed line in Fig. 4d). Representative image of Alcian blue and Sirius red-stained section of the host articular cartilage with partial thickness defect after the 16 week culture period without the neocartilage implant demonstrated absence of cartilage regeneration (Fig. 4e).

## 4. Discussion

The present study represents the first successful application of the novel microfluidic perfusion bioreactors with integrated USWT to bioengineer robust, 3-D, scaffold-free neocartilage grafts of HACs that are analogous to native hyaline cartilage. Additionally, in an *ex vivo* organ culture model, the study has demonstrated the potential of the neocartilage grafts to mediate repair of partial thickness chondral defects by generation of hyaline cartilage-like repair tissue. A scaffold-free tissue engineering approach was employed by the present study, instead of a scaffold-based approach, for the *ex vivo* generation of 3-D cartilage constructs. This is because, in comparison to the natural extracellular matrix, which aids cartilage regeneration by providing crucial cues to chondrocytes, some artificial scaffold materials may impair tissue formation and defect regeneration due to their unpredictable degradation rates and immunogenicity of the degradation products.^44^ While the pellet culture technique is an effective *ex vivo* scaffold-free strategy to stimulate chondrogenic redifferentiation of dedifferentiated chondrocytes and promote cartilage formation in a high cell density 3-D microenvironment,^45^ it has limited clinical application because conventional static pellet culture is associated with lack of mechanical stimulation, inefficient oxygen diffusion and suboptimal metabolite mass transfer rates, which adversely affect the scale-up, quality (*i.e.* formation of fibrous *versus* hyaline cartilage) and biomechanical properties of the chondrospheres. This was demonstrated in our previous study, where attempts to scale-up the size of constructs by conventional static pellet culture using 1 × 10^6^ HACs, resulted in the formation of chondrospheres that were characterised by the development of necrotic cores and suboptimal hyaline cartilage.^46^ Mass transport limitations and oxygen diffusion gradients in the macroscopic pellets have been recognised as significant obstacles to robust chondrogenesis, thereby limiting the prospects of macroscopic cartilaginous pellets/chondrospheres in the repair of chondral defects.^47^

The scaffold-free grafts bioengineered in the acoustofluidic perfusion bioreactors using 1 × 10^6^ HACs exhibited distinct hyaline cartilage-like tissue, which was characterised by the robust expression of chondrogenic markers, namely SOX-9, type II collagen and proteoglycans, coupled with negligible expression of collagen types I and X. Previous studies have demonstrated induction of type II collagen and proteoglycans by human articular chondrocytes in 3-D alginate culture in response to low intensity ultrasound, and suppression of chondrocyte hypertrophy by inhibition of expression of type X collagen due to low intensity ultrasound treatment.^48,49^ Although the exact mechanism by which ultrasound promotes the expression of chondrogenic markers remains to be fully elucidated, it has been suggested that excitation of microbubbles or acoustic streaming produced by the ultrasound can modulate mechanoreceptor-mediated transmembrane signalling mechanisms (involving protein kinase C) for the regulation of *Aggrecan* gene expression and stimulation of subsequent proteoglycan synthesis.^50–52^ Moreover, both cell shape and cytoskeletal organisation were shown to be essential for initiation of Sox-9 expression and maintenance of the differentiated chondrocyte phenotype in 2-D aggregates of chick wing bud mesenchymal cells, which were generated and levitated using an USWT.^53^

To enhance mass transfer and mechanical stimulation, the current system employed two strategies, namely continuous perfusion of the culture medium at rates considered low-shear and sweeping acoustic drive frequencies over the range of 890 to 910 kHz, at a sweep rate of 50 Hz. The sweep rate of 50 Hz reflected the maximum value available from the signal generator that was used in the study. The application of fluid shear from the perfusion system in our device compensated for the reduced acoustically-induced forces exerted on the cells by the ultrasonic waves. Mechanical stimulation conveyed by the flow of the culture medium *i.e.* fluid flow-induced shear, has been acknowledged as a crucial biomechanical stimulus, which enhances chondrocyte function and *ex vivo* cartilage formation by directly influencing cell metabolism and extracellular matrix synthesis.^13^ Moreover, chondrocytes respond positively to fluid convection, which has been shown to promote the transport of molecules and further improve cartilage formation due to enhanced mass transfer rates of metabolites.^54^

As a significant departure from previous studies that applied either low intensity pulsed ultrasound (1–1.5 MHz, 1 kHz repeat, 6–40 min) or intermittent low intensity diffuse ultrasound (5 MHz excitation frequency) to stimulate chondrocytes,^20,29,51^ in our study the ultrasound was constantly applied over the 21 day culture period and swept over a small range of frequencies, thereby exciting a range of standing wave resonances in the capillary. Initially, the frequency sweep was applied in order to facilitate multiple devices (that were hand-assembled and hence, exhibited slight differences in resonant frequencies) to be run simultaneously from a single amplifier, and to allow for small changes in the resonant frequency with time due to temperature fluctuations and/or geometric changes of the transducers as a result of autoclaving. The range (890–910 kHz) was chosen so as to include the half-wavelength resonant frequencies of the three devices (897, 899 and 902 kHz). However, an additional effect is observed that warrants further investigation in the future: the frequency sweeping caused the agglomerates to vibrate/oscillate at the sweep frequency of 50 Hz, resulting in the application of additional fluidic shear stresses to the agglomerates. This effect was more easily observed at lower sweep frequencies, at which it was apparent that each individual frequency of the sweep range created a slightly different resonance and related trapping position. At lower sweep frequencies *e.g.* when the sweep frequency was reduced to 1 Hz, the cell agglomerates were observed to oscillate with ~1 mm vibration amplitude. Based on these preliminary observations, we hypothesise that the mechanical stimulation provided by the vibration applied to the cell agglomerates, in addition to the fluid shear from continuous perfusion of the chondrogenic culture medium, may be crucial for stimulating robust chondrogenesis and hyaline cartilage formation in the agglomerates. Further work is however required to optimise the sweep rate, quantify the mechanical forces acting on the agglomerates and examine their effects in detail on cartilage formation by the cells of the agglomerates.

When ultrasound is absorbed by a material, its mechanical energy is primarily converted into heat. The acoustofluidic perfusion bioreactors in the present study are custom-built using glass capillaries and filled with culture medium, both of which are ‘low-loss materials’. Moreover, the capillary construction limits transmission of the ultrasound into the surrounding structure, thereby resulting in a high resonant Q-factor. Hence, it is possible to generate a substantial acoustic field without significant heating.^55^ The steady state temperature rise recorded by the thermocouple embedded within our device was 0.8 °C. Additionally, in the current experimental setup, a custom made incubator incorporating a fan and thermostatic control maintained the environmental temperature at around 36 °C, thus ensuring the optimal cell culture temperature close to 37 °C in the active region of the bioreactor. Prolonged exposure to ultrasound in the acoustofluidic perfusion bioreactor therefore did not adversely affect cell viability, as confirmed by the presence of metabolically active viable cells and absence of necrotic cells in day 21 neocartilage explants.

To determine the micromechanical properties of the neocartilage grafts, elastic moduli of the bulk material of day 21 grafts were measured using an IT-AFM microtip (spherical indenter diameter of 10 μm), and compared to the elastic moduli of freshly isolated full-thickness human articular cartilage pieces. Previous work has reported that the microscale elastic modulus of human articular cartilage, determined using IT-AFM, is 1.3 MPa regardless of the degree of OA, while changes due to aging and/or OA are only depicted at the nanometer scale.^43^ Harnessing a similar IT-AFM setup, we obtained comparable value for elastic modulus [1.34 ± 0.315 MPa] of the full-thickness human articular cartilage pieces utilised in the present study. Interestingly, the elastic modulus [0.90 ± 0.372 MPa] of day 21 neocartilage grafts was similar to the elastic modulus of human articular cartilage. Thus, the neocartilage constructs were not only histologically comparable to hyaline cartilage, but also displayed comparable mechanical competency as native articular cartilage.

An *ex vivo* organ culture partial thickness cartilage defect model was utilised in the present study to determine the ability of the neocartilage explants to integrate with native human articular cartilage and repair the defects. Integration is defined as the absence of gaps between the surface of the repair tissue generated by the cartilage graft and the border of the native cartilage matrix in the defect region.^56^ Often after implantation, cartilaginous grafts do not integrate readily or predictably with the host tissue to form a continuous mechanically stable attachment. This largely occurs because the repair tissue is predominantly composed of fibrocartilage, which is deficient in proteoglycans and mechanically inferior, compared to the host hyaline cartilage.^57^ In the present study, following implantation of the neocartilage graft into the chondral defect and co-culture for 16 weeks *ex vivo*, the defect was filled with hyaline cartilage-like tissue characterised by the presence of numerous chondrocytes embedded in dense proteoglycan matrix. Although a discernible boundary was visible, no gaps were observed between the edge of the repair tissue and the border of the defect in the host cartilage. This was indicative of continuous close attachment between the newly synthesized hyaline cartilage-like repair tissue and the host articular cartilage, and integration of the repair tissue into the surrounding native cartilage. Thus, implantation of the neocartilage graft into the chondral defect resulted in the generation of hyaline cartilage-like repair tissue that contributed to significant improvements to the tissue architecture within the defect, in comparison to absence of cartilage regeneration in the empty defect. Further investigations will focus on examining the biomechanical properties of the repair tissue.

We acknowledge the limitations of the *ex vivo* organ culture partial thickness cartilage defect model used in our study to reproduce the complex biological and mechanical environment of the joint. Since constructs demonstrate a certain threshold of function *in vitro*, to carry out a realistic assessment of their potential for cartilage repair, we recognise that they should be assayed in a large animal (*e.g.* lapine model) load-bearing environment.^9^ Future work will therefore involve implantation of the neocartilage grafts in partial thickness chondral defects created in rabbit lateral femoral condyles and long-term assessment of the repair tissue post-implantation.

## 5. Conclusions

In summary, we have successfully applied a novel tissue engineering approach that combines microfluidic perfusion bioreactor technology with continuous application of ultrasound to bioengineer scaffold-free (hence devoid of any foreign material) neocartilage grafts, which are analogous to native hyaline cartilage both histologically and biomechanically, and have the ability to repair partial thickness chondral defects. The work therefore presents a real opportunity to derive a robust tissue-based product, which has the potential for subsequent use in restorative approaches, such as II generation ACI, for the repair of focal partial thickness chondral defects in early stage OA.
